# Supplementary material for: Novel Insights on the Role of Epigenetics in Androgen Receptor’s Expression in Prostate Cancer
Source: Biomolecules. 2023 Oct 14;13(10):1526. doi: 10.3390/biom13101526 (PMC10605369; doi:10.3390/biom13101526)
Supplement: Supplementary file 1 [file biomolecules-13-01526-s001.zip › biomolecules-2606864-supplementary/biomolecules-2606864-supplementary.pdf]

biomolecules

MDPI

Supplementary Data

Novel insights on the role of epigenetics in androgen receptor's expression in prostate cancer

Vânia Camilo [1+], Mariana Brütt Pacheco [1+] and Filipa Moreira-Silva [1]; Gonçalo Outeiro-Pinho [1]; Vítor M. Gaspar [2]; João F. Mano [2]; C. Joana Marques [3; 4]; Rui Henrique [1; 5; 6]; Carmen Jerónimo [1; 6; *]

Supplementary Figures

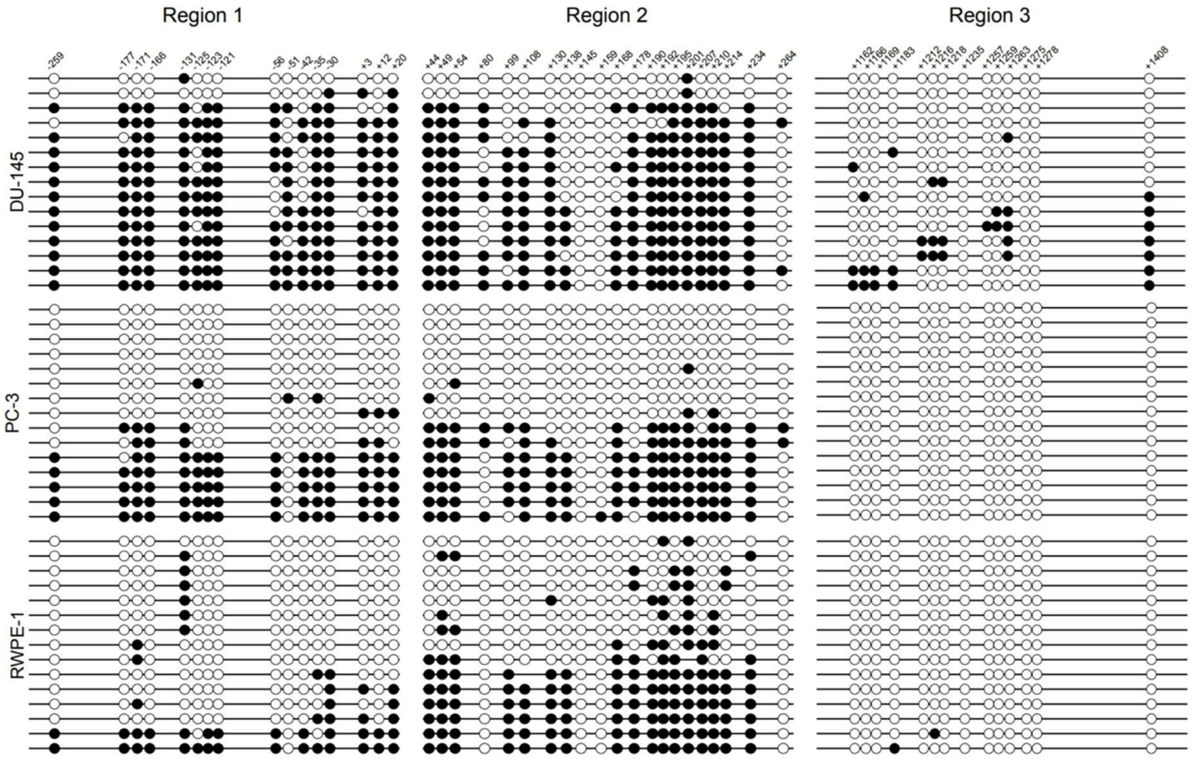

Figure S1. Specific CpG methylation status of *AR* regions 1 to 3 in WT prostate cell lines. DU-145 (upper), PC-3 (middle) and RWPE-1 (lower) cell lines' methylation was assessed in 15 independent clones. Each row represents one clone, and the presence or absence of methylation is represented by a black or white circle, respectively. The clones are ranked from top to bottom according to the number of methylated CpGs.

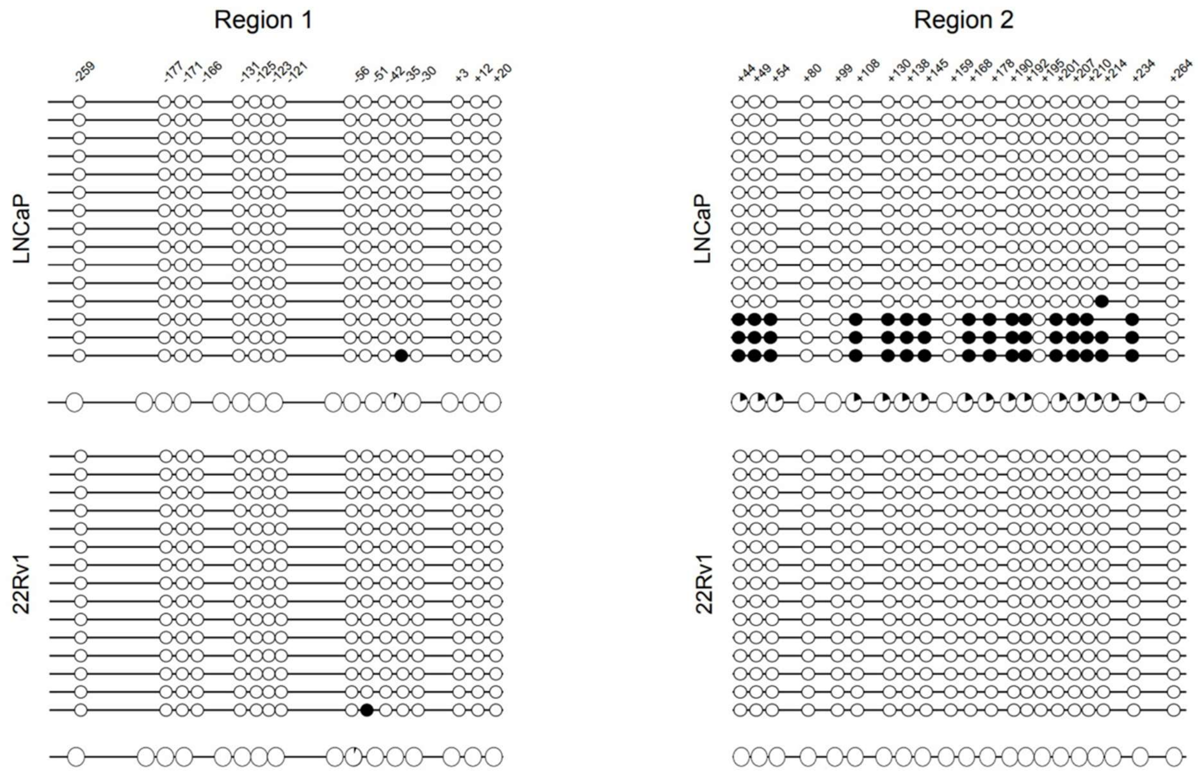

Figure S2. Specific CpG methylation status of *AR* regions 1 and 2 in AR positive PCa cell lines. LNCaP and 22Rv1 cell lines' methylation was assessed in 15 independent clones. Each row represents one clone, and the presence or absence of methylation is represented by a black or white circle, respectively. The clones are ranked from top to bottom according to the number of methylated CpGs. The mean percentage of methylation per region is displayed under the sequences of individual clones.

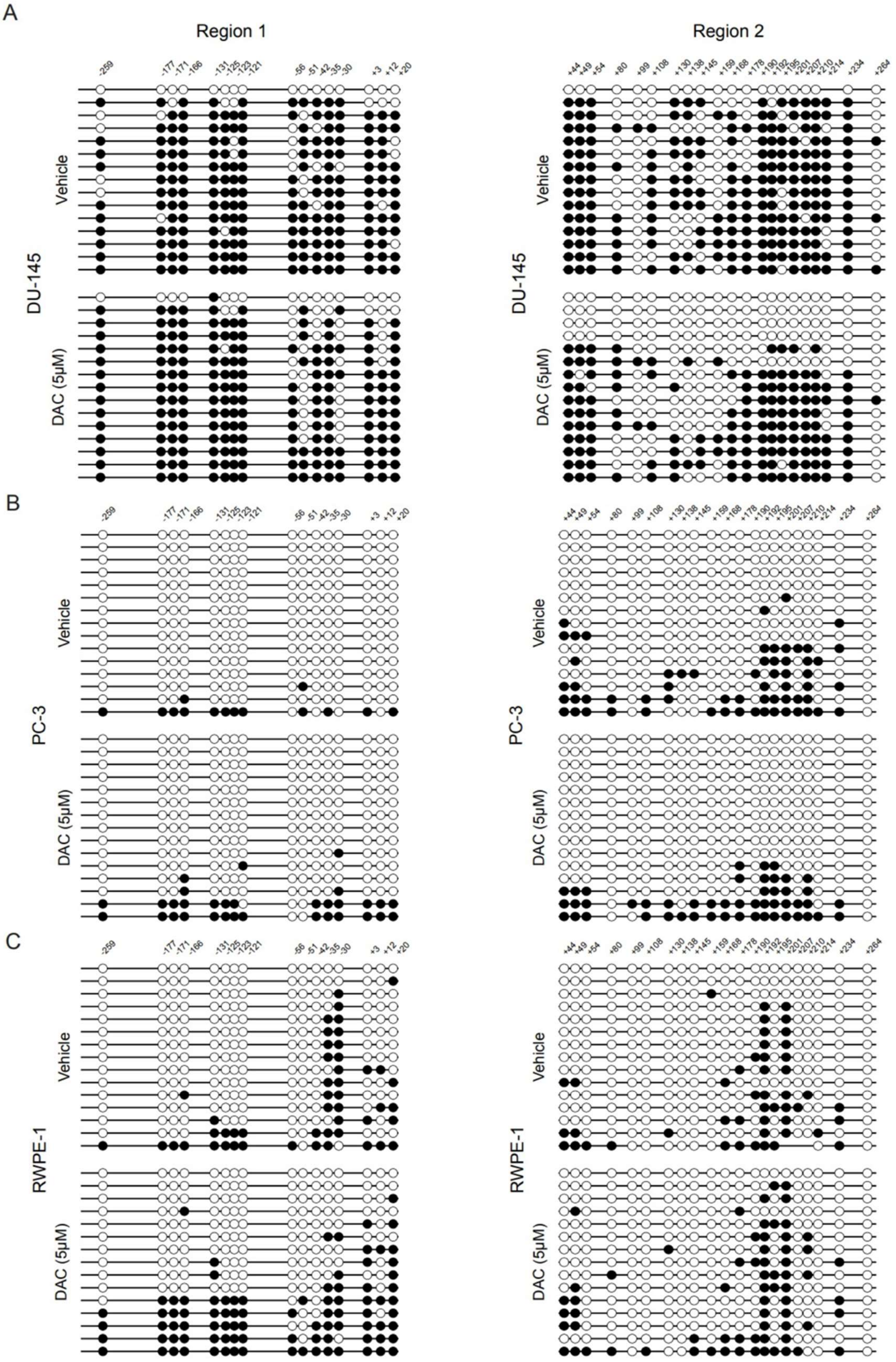

Figure S3. Specific CpG methylation status of *AR* regions 1 and 2 in prostate cell lines following treatment with DAC. DU-145 (**A**), PC-3 (**B**) and RWPE-1 (**C**) cell lines' methylation was assessed in 15 independent clones following exposure to DAC at 5μM or its respective vehicle control. Each row represents one clone, and the presence or absence of methylation is represented by a black or white

circle, respectively. The clones are ranked from top to bottom according to the number of methylated CpGs.

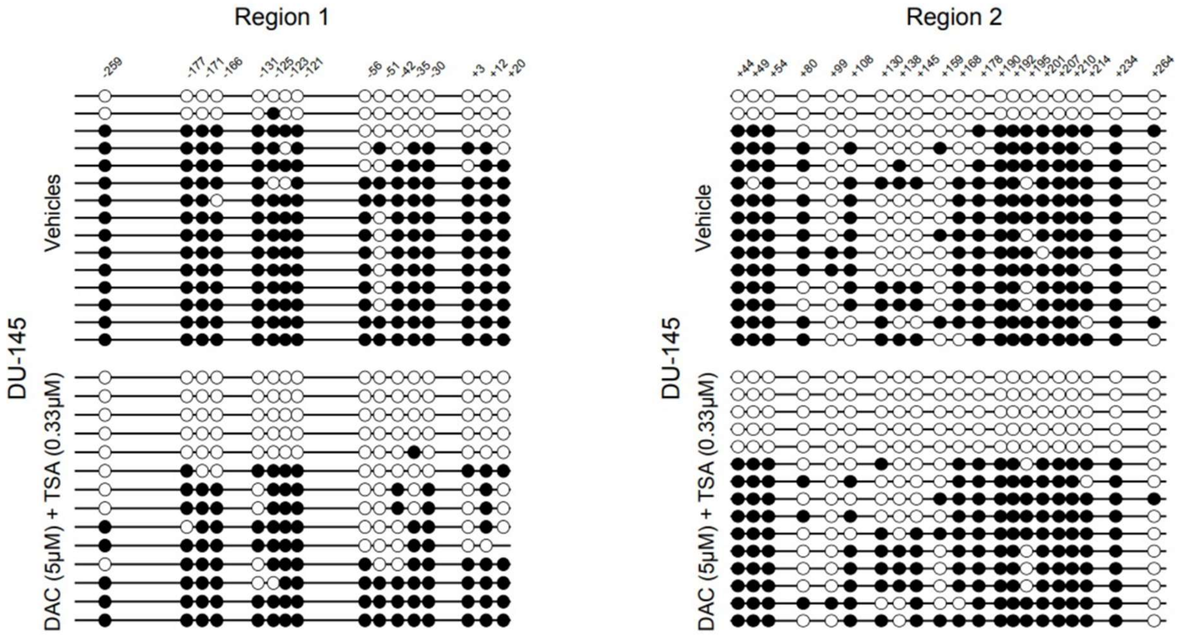

Figure S4. Specific CpG methylation status of *AR* regions 1 and 2 in DU-145 cells following combined treatment with DAC and TSA. DU-145 methylation was assessed in 14 or 15 independent clones following exposure to DAC at 5µM and TSA at 0.33µM or vehicle control, respectively. Each row represents one clone, and the presence or absence of methylation is represented by a black or white circle, respectively. The clones are ranked from top to bottom according to the number of methylated CpGs.

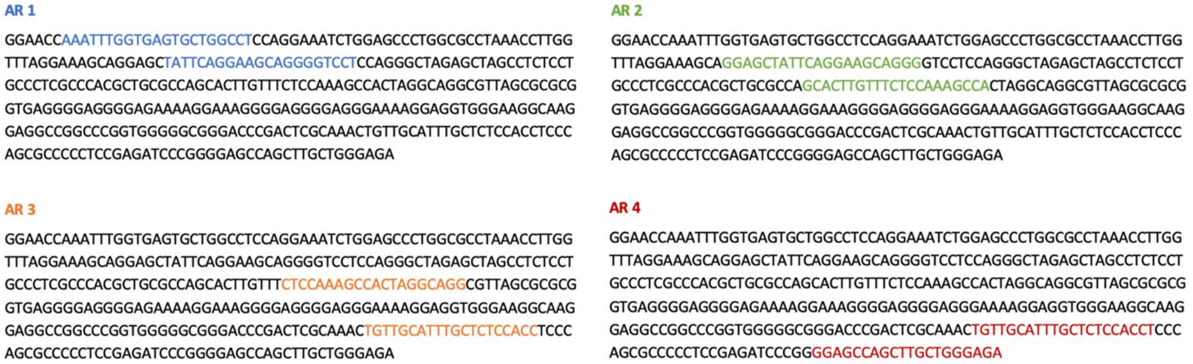

Figure S5. Localization of AR1-4 primers, within *AR* region 1, used in Chip-PCR analysis. Primers AR1, AR2, AR3 and AR4 localization within *AR* region 1.

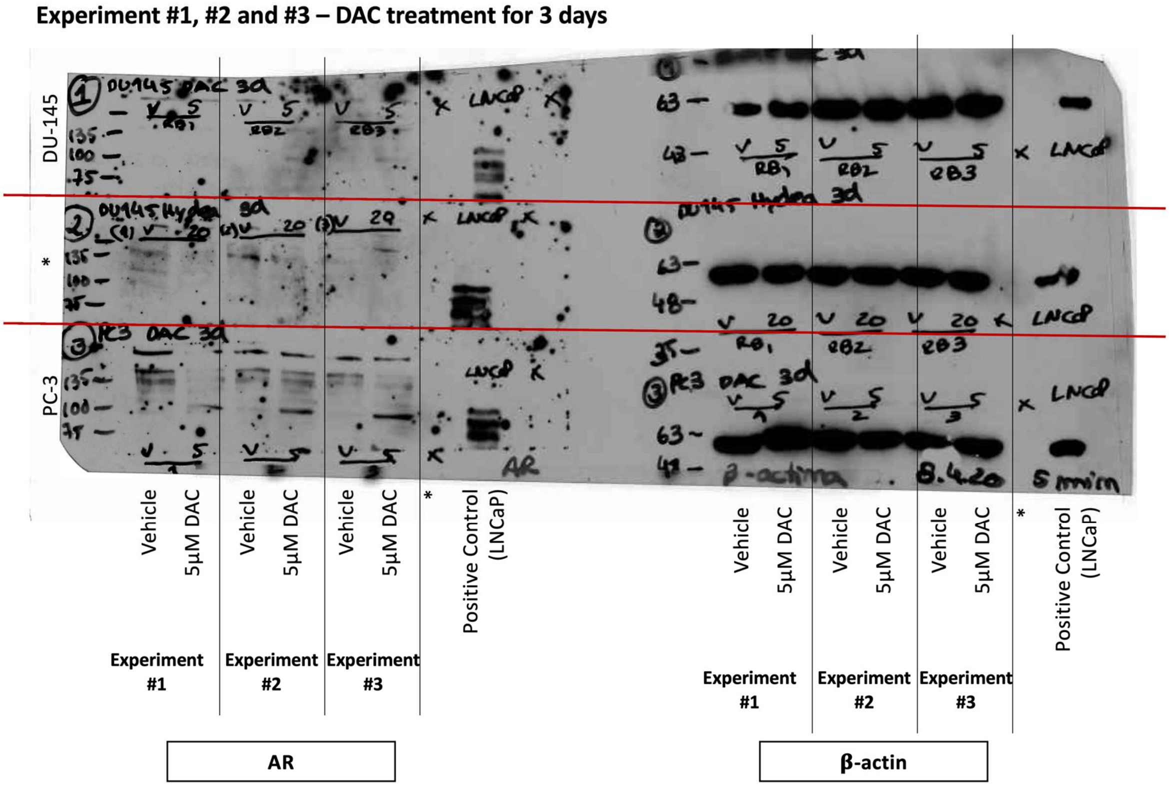

Figure S6. Original Western Blots of the experiments used for the representation of the proteins presented in Figure 2D. The symbol * represents lanes and experiments that were not used for the representation of the proteins presented in Figure 2D.

Experiment #1, #2, #3 and #4 – DAC and TSA treatment for 3 days, alone and in combination

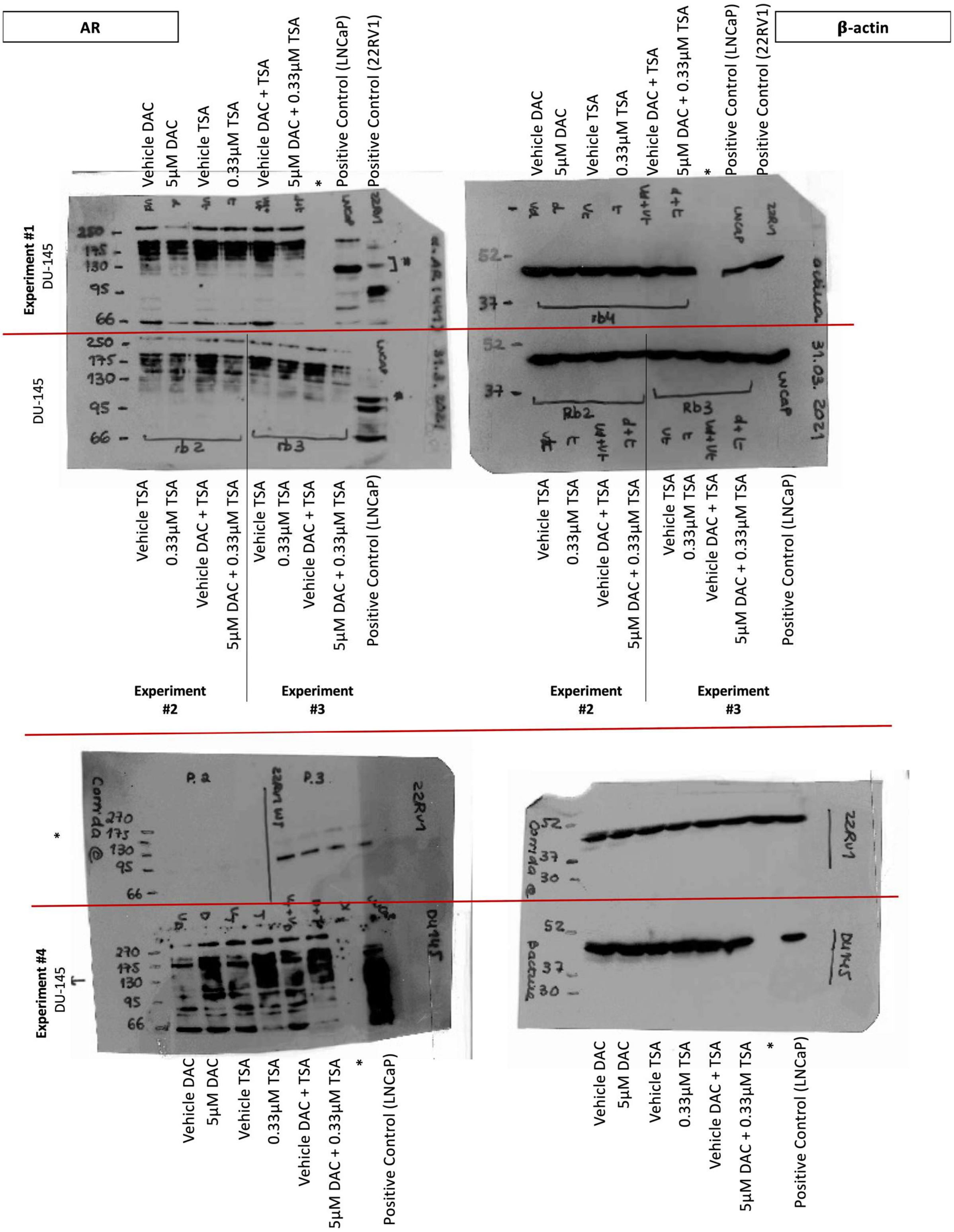

Figure S7. Original Western Blots of the experiments used for the representation of the proteins presented in Figure 3C. The symbol * represents lanes and experiments that were not used for the representation of the proteins presented in Figure 3C.

Supplementary Tables

Table S1. List of antibodies used in this study.

Antibody	Species	Dillution	Blocking	Clone	Supplier	Positive Control	Expected MW
AR	Mouse (mAb)	1:200	5% NFDM/TBS-T	AR-441	Sigma Aldrich, USA	LNCaP	110kDa
β-actin	Mouse (mAb)	1:10,000	5% NFDM/TBS-T	A1978	Sigma Aldrich, USA	n.a	42KDa

mAb – monoclonal antibody; NFDM – non-fat dry milk; TBS-T- Tris Buffer Saline, 0.1% Tween-20; n.a – not applicable; MW – molecular weight.

Table S2. List of primers used in this study.

Gene	Primer Forward (5′-3′)	Primer Rev (5′-3)	$T_{annealing}$
AR _region 1 (BSP)	GGAATTAAATTTGGTGAGTGT	TCTCCCAACAAACTAACTCC	60ºC
AR_region 2 (BSP)	GGAGTTAGTTTGTTGGGAGA	CCTACCAAACACTTTCCTTAC	60ºC
AR_ region 3 (BSP)	AAGTTTAAGGATGGAAGTGTAGTT	TTACTATTCCTCATCCAGGACC	60ºC
miR-130a (qMSP)	ATAAATTTTGTCGGGGAGAGC	AATACCCCGATCAACGAAAA	64ºC
CCND2 (qMSP)	TTTGATTTAAGGATGCGTTAGAGTACG	ACTTTCTCCCTAAAAACCGACTACG	62ºC
RASSF1A(qMSP)	GGGTTTTGCGAGAGCGCG	GCTAACAAACGCGACCG	60ºC
ACTB (qMSP)	TGGTGATGGAGGAGGTTTAGTAAGT	ACCAATAAAACCTACTCCTCCCTTAA	60ºC
AR 1 (ChIP)	GGAGCTATTCAGGAAGCAGGG	GCACTTGTTTCTCCAAAGCCA	60ºC
AR 2 (ChIP)	CTCCAAAGCCACTAGGCAGG	TGTTGCATTTGCTCTCCACC	60ºC
AR 3 (ChIP)	AAATTTGGTGAGTGCTGGCCT	TATTCAGGAAGCAGGGGTCCT	60ºC
AR 4 (ChIP)	TGTTGCATTTGCTCTCCACCT	AGGCGACAGAGGGAAAAAGG	60ºC

BSP- Bisulfite Sequencing Primer; qMSP- Quantative Methylation Specific Primer; ChIP – Chromatin Immunoprecipitation
